# Predictive factors of inadequate bowel preparation for elective colonoscopy 

**Published:** 2022

**Authors:** Amir Sadeghi, Mohsen Rajabnia, Mohammad Bagheri, Shaghayegh Jamshidizadeh, Samane Saberi, Paria Shahnazi, Leila Pasharavesh, Mohamad Amin Pourhoseingholi, Mona Mirzaei, Hamid Asadzadeh Aghdaei, Mohammad Reza Zali

**Affiliations:** *Gastroenterology and Liver Diseases Research Center, Research Institute for Gastroenterology and Liver Diseases, Shahid Beheshti University of Medical Sciences, Tehran, Iran*

**Keywords:** Colonoscopy, Colon cleaning, Bowel preparation, Risk factors of bowel cleansing, Quality of colonoscopy

## Abstract

**Aim:**

This study aimed to evaluate the effects of factors like demographic items, comorbidities, and drug history on the inadequacy of colonic preparation before colonoscopy.

**Background:**

Inadequate bowel preparation can lead to lower polyp detection rates, longer procedure times, and lower cecal intubation rates.

**Methods:**

This population-based study was conducted on 2476 Iranian adults who were referred to two tertiary centers for elective colonoscopy between 2017 and 2018. Bowel preparation quality was scored by the Boston bowel preparation scale (BBPS). Univariate and multivariate logistic regressions were used to find the independent predictors of bowel preparation inadequacy.

**Results:**

The results showed that 31.8% of patients had inadequate bowel preparation before their colonoscopy. Higher age, BMI>25, abdominal circumference>95 cm, low fruit consumption, and history of smoking were independently correlated with bowel preparation inadequacy. Additionally, using NSAIDs and SSRIs were correlated with bowel preparation adequacy in multivariate regression analysis. Finally, age, gender, ethnicity, BMI, abdominal circumference, fruit consumption, smoking, NSAIDs, SSRIs, education, constipation, physical activity, and diabetes entered the predictive model of this study. The area under the curve (AUC) reached 0.70 in the final step.

**Conclusion:**

The independent risk factors associated with colonic preparation inadequacy were identified, and herein, a predictive model is suggested for identifying patients with a high risk of bowel preparation inadequacy before a colonoscopy so that alternative preparation techniques can be employed among high-risk groups to yield optimal preparation quality.

## Introduction

 Colonoscopy has long been considered the gold standard for colorectal cancer (CRC) screening because of its capabilities in exploring the colon and removing colorectal polyps that could turn cancerous in the future (1). It is critical to have an appropriate bowel preparation before performing a colonoscopy examination to achieve a high quality, effective, and safe procedure (2). In addition, suitable colonoscopy preparation must effectively clean the lumen, while having no histological adverse effects on colonic mucosa (3-5). 

Inadequate bowel preparation can result in failure to detect polyps equal to or larger than 5 mm in size and increase the risk of procedural adverse events, such as bleeding or perforation (3). Moreover, insufficient bowel preparation can lead to lower detection of polyps and adenomas of various sizes (6, 7), longer overall procedure time, more frequent repetitions of colonoscopies, higher risk of perforation, longer CIT, prolonged hospitalization, and higher costs of procedures (3, 8, 9). Cecal intubation time (CIT) is defined as the time between insertion of the colonoscope into the anus and when the colonoscope tip passes to a point proximal to the ileocecal valve in which the base of the cecum is visible. Actually, in clinical practice, longer CITs can be seen in patients with inadequate preparation (10). Therefore, inadequate preparation reduces the quality of colonoscopy and increases the likelihood of lesions and evidence of disease (such as mucosal changes suggesting ulcerative colitis) not being identified; this delay in diagnosis and, consequently, lack of timely treatment will lead to the progression of the disease and its complications.

A lack of proper preparation can also increase the risk of complications from the procedure, morbidity and mortality, length of hospital stay, and the cost of treatment.

Previous studies have reported that 17.2% to 44.2% of colonoscopies were performed under inadequate preparation conditions (11-16). Furthermore, some authors have declared that various demographic factors, socioeconomic factors, previous comorbidities, and drug history may affect the quality of colonoscopy preparation (17, 18). Accordingly, several studies have evaluated the factors associated with bowel preparation quality (16, 18-20); however, only a small fraction of them has proposed a predictive model to identify those patients with a high risk of bowel preparation inadequacy before a colonoscopy (13, 21, 22). Therefore, the current study evaluated the effects of some factors, i.e. demographic items, comorbidities, and drug history, on the inadequacy of colonic preparation before colonoscopy. Accordingly, a predictive model is suggested for identifying patients with a high risk of bowel preparation inadequacy before colonoscopy so that alternative preparation techniques can be employed and the cleansing regimen intensified so as to yield optimal preparation quality. 

## Methods

**Participants**

This study was conducted on Iranian adults who referred to Taleghani Hospital, which is a gastroenterology university center in Tehran, Iran, for elective colonoscopy during 2017 and 2018. All patients shared similar socio-economic statuses (SES). This study was conducted in accordance with the principles of the Declaration of Helsinki and approved by the Research Ethics Committee of the Research Institute for Gastroenterology and Liver Diseases, Shahid Beheshti University of Medical Sciences. Informed written consent was obtained from each subject.

**Data collection**

Information on the demographic, anthropometric and lifestyle features, socioeconomic status, and medical and family histories was collected for each of the patients in a visit before a colonoscopy. Educational level was categorized as being illiterate or literate (primary school or higher), low fruit consumption from low (never use to weekly use) to high (daily use), vegetable consumption from low (not eating to weekly use) to high (daily use), smoking as never and ever (smoking now or was a smoker and quit), physical activity as never (never or less than 30 minutes in a day, weekly) and ever (regular exercise or more than 30 minutes per day/weekly), and ethnicity as Fars or other Iranian ethnic group.

**Colonoscopy preparation method**

The preparation regimen of the patients scheduled for a morning colonoscopy was comprised of six liters of polyethylene glycol (PEG) solution and three bisacodyl tablets (FDA-approved regimen, i.e. PEG-containing regimen) one day before the colonoscopy. Patients undergoing an evening colonoscopy consumed five liters of PEG and three bisacodyl tablets the day before the procedure, and another one liter in the morning of the day of the procedure. A liquid-based regimen was started the day before the procedure, and having proper physical activity in the preparation period was recommended. All patients received preparation instructions at their appointment in the clinic. The preparation regimen, the importance of bowel preparation, and the effect of diet modification were described. A brochure containing instructions was given to all patients.

**Evaluation of bowel preparation**

Colonoscopies were performed by three expert colonoscopists, who performed an annual minimum of 400 colonoscopies each year. Fentanyl and midazolam were used for moderate conscious sedation during the procedure. Reaching the cecum and ileocecal valve by colonoscope tip was considered the end of the examination. Colonic preparation was evaluated in the whole colon and in its three anatomic parts separately, as follows: ascending, descending, and transverse. Bowel preparation quality was scored by the Boston bowel preparation scale (BBPS), a valid and reliable measure of bowel preparation that reflects the colon’s cleanliness during the inspection phase of the procedure (23). Each segment of the colon is scored between 0 (unprepared colon) to 3 (fully prepared colon), 0-1 is considered as inadequate and 2-3 as adequate preparation. Finally, the sum of each three segments score created the BBPS score, in which BBPS score 0-5 was considered as an inadequate bowel preparation and 6-9 (certainly preparation of any segment should not be less than 2) as an adequate preparation. 

**Statistical analysis**

Continuous and categorical variables are described as mean (SD) and number (%), respectively. The distribution of variables that were significantly different between adequately and inadequately prepared individuals was forced into the regression models. Thereafter, the candidate variables were examined based on their univariable association with the outcome, and then, seven significant preparation indicators, i.e. BMI, abdominal circumference, education, fruit consumption, vegetable consumption, smoking, and physical activity, were combined to generate a variable with all possible configurations. Moreover, multivariable logistic regression models were used to estimate the odds of having inadequate colon preparation, given the “combined variable” as the risk indicator of interest. Multivariable linear regression that treats variables as independent variables was done to evaluate the roles of demographic variables, diseases, and drug consumption on colon preparation. While the effect of one demographic variable in these models was of interest, other demographic variables were considered as potential confounders and remained in the model. The full model was built with the covariate effects using the stepwise inclusion method. Then, the significance of covariates was tested in the full model using the backward elimination method. The Akaike information criterion (AIC) and Bayesian information criterion (BIC) were computed each time a variable was included/excluded, and the model with the lower AIC or BIC value was preferred. Additionally, the interactions of age, gender, ethnicity, and other predictors in the model were tested and model performances determined by examining measures of calibration and discrimination. Calibration refers to how closely the predicted probability of having inadequate colon preparation agrees with the observed inadequacy status (24), assessed by the Hosmer-Lemeshow test. Discrimination refers to the ability of the clinical decision rule to differentiate between individuals with and without adequate colon preparation, as measured by the area under the receiver operating characteristic (ROC) curve (AUC) statistic. In this study, an AUC value of 0.5 is described as no discrimination and a value of one is perfect discrimination. Stata software (version 14) was used for analyses, and the results were considered as statistically significant at a *p*=0.05 levels. 

## Results

**Baseline characteristics:**

**Table 1:** Baseline characteristics of patients enrolled in this study

	Studied Patients n=2476 (%)
BMI (Mean SD)^1^	26.40 5.22
Median/ Rang (Min, Max)	26.03/ 59.59 (13.06, 72.65)
Abdominal circumference (Mean SD)^2^	90.52 12.34
Median/ Rang (Min, Max)	90.00/ 78.00 (62.00, 140.00)
Indication of colonoscopy	
FIT positive	288 (11.6)
Family history of polyps	887 (35.8)
Abdominal pain	719 (29.0)
Constipation	261 (10.5)
Other reasons	321 (13.1)
Fruit consumption	
High	1590 (65.1)
Never or Low	852 (34.9)
Vegetable consumption	
High	1065 (44.1)
Never or Low	1350 (55.9)
Smoking	
Never	2064 (84.4)
Ever	382 (15.6)
Physical Activity	
Never	531 (21.9)
Ever	1894 (78.1)
PEG consumption (Mean SD)^3^	
Adequate	5.15 1.25
Inadequate	5.07 1.51
Cecum Intubation time (Mean SD)^4^	299.5 155.8
Median/ Rang (Min, Max) Adequate inadequate	240.0/ 969.0 (120.0, 1089.0)
	263.5 95.5
	349.8 203.7
Preparation quality by BBPS	
Adequate	1688 (68.2)
Inadequate	788 (31.8)
Preparation quality of ascending colon	
Adequate	1820 (73.5)
Inadequate	656 (26.5)
Preparation quality of transverse colon	
Adequate	1817 (73.4)
Inadequate	659 (26.6)
Preparation quality of descending colon	
Adequate	1782 (72.0)
Inadequate	694 (28.0)

A total of 2476 participants aged between 18 and 80 years were enrolled in the current study. The mean age of the study participants was 51.32 14.80 years. Out of all participants, 1407 (56.82%) were female, and the ethnicity of 1314 (53.2%) of them were Fars. The analyses showed that 68.2% of participants had adequate bowel preparation measured by BBPS score, and 85.7% of procedures were conducted in the evening. No significant difference was observed in the amount of PEG consumption between the patients with adequate and those with inadequate bowel preparation or in the prevalence of preparation inadequacy between all three parts of the colon (Table 1). CIT was registered in a subgroup of patients (n=228), in which it was calculated as 263.5 95.5 s in patients with adequate bowel preparation and 349.8 203.7 s in patients with inadequate bowel preparation, indicating a higher CIT in the inadequate bowel preparation group compared to those with adequate preparation (*p*<0.001) (Table1). 

**Univariate analysis of risk factors**

The univariate analysis of demographic, anthropometric, and clinical characteristics of participants in the current study is summarized in Table 2. The results showed that bowel preparation inadequacy measured by BBPS was significantly correlated with higher age, higher BMI, larger abdominal circumference, illiteracy, lower fruit consumption, lower vegetable consumption, lower physical activity, and smoking (*p*<0.05). Moreover, diabetes and constipation were other conditions that significantly affected the colonic preparation and were correlated with the inadequacy of bowel preparation by BBPS (*p*<0.05). Other variables, including gender, ethnicity, IBD, and coronary heart disease, were not associated with colon preparation quality. Furthermore, it was found that ASA, NSAIDs, SSRI, PPI, gliclazide, insulin, metformin, vitamin D3, ACEI or ARB, GnRH, and calcium were significantly correlated with the improvement of bowel preparation adequacy by BBPS score (*p*<0.05). The association between use of various drugs and colonic preparation is presented in Table 3.

**Table 2:** Demographic, anthropometric, and clinical features and their relationship to colonic preparation in the studied Population

		BBPS score			
		Adequate 1688 (68.2)	Inadequate 788 (31.8)	OR (95% CI)^*^	P-value
Age					
Mean SD		50.44 14.57	53.22 15.12	1.01 (1.01- 1.02)	<0.0001
Gender					
Female		979 (58.0)	428 (54.3)		
Male		709 (42.0)	360 (45.7)	1.16 (0.98- 1.38)	0.085
Ethnicity					
Fars		904 (53.6)	410 (52.2)		0.510
Other ethnic groups		781 (46.4)	375 (47.8)	1.06 (0.89- 1.25)	
BMI					
Mean SD		25.51 4.10	28.16 6.57	1.11 (1.09- 1.13)	<0.0001
Abdominal circumference					
Mean SD		88.68 11.65	94.17 12.84	1.04 (1.03- 1.04)	<0.0001
Education					
Educated		1507 (89.3)	664 (84.6)	1.53 (1.19- 1.95)	0.001
Illiterate		180 (10.7)	121 (15.4)		
Fruit consumption					
High		1195 (71.3)	394 (51.6)	2.33 (1.95- 2.78)	<0.0001
Never/Low		482 (28.7)	370 (48.4)		
Vegetable consumption					
High		788 (47.0)	276 (37.4)	1.49 (1.24- 1.78)	<0.0001
Never/Low		887 (53.0)	462 (62.6)		
Smoking					
Never		1431 (85.8)	633 (81.4)	1.39 (1.11- 1.74)	0.005
Ever		236 (14.2)	145 (18.6)		
Physical activity					
Never		383 (23.2)	149 (19.3)	1.26 (1.02- 1.56)	0.030
Ever		1268 (76.8)	624 (80.7)		
IBD					
No		1527 (92.4)	719 (94.4)	0.72 (0.51- 1.04)	0.077
UC^1^		111 (6.7)	33 (4.3)		
Yes CD^2^		12 (0.7)	9 (1.2)		
IC^3^		3 (0.2)	1 (0.1)		
Diabetes					
No		1561 (94.2)	691 (91.3)	1.55 (1.12- 2.15)	0.008
Yes		96 (5.8)	66 (8.7)		
Coronary heart disease					
No		1554 (93.8)	694 (92.3)	1.26 (0.90- 1.76)	0.174
Yes		103 (6.2)	58 (7.7)		
Constipation					
No		1031 (60.8)	442 (57.1)	1.20 (1.00-1.43)	0.045
Yes		652 (39.2)	332 (42.9)		


* OR is reported for inadequate groups compared with adequate groups as reference group. 1 UC, Ulcerative colitis. 2 CD, Crohn’s disease. 3 IC, indeterminate colitis. Data adjusted for age, gender, and ethnicity

**Table 3:** Drug consumption features and their relationship to colonic preparation in the studied population

		BBPS score			
		Adequate 1688 (68.2)	Inadequate 788 (31.8)	OR (95% CI)^*^	P-value
ASA	No	1415 (85.3)	626 (81.4)		
	Yes	243 (14.7)	143 (18.6)	1.33 (1.06- 1.67)	0.014
NSAID	No	1565 (94.6)	744 (97.5)		
	Yes	89 (5.4)	19 (2.5)	0.45 (0.27- 0.74)	0.002
Anti-TNF	No	1638 (99.0)	755 (98.8)		
	Yes	16 (1.0)	9 (1.2)	1.22 (0.54- 2.77)	0.635
Prednisolone	No	1637 (99.0)	756 (99.0)		
	Yes	17 (1.0)	8 (1.0)	1.02 (0.44- 2.37)	0.965
Azathioprine	No	1628 (98.4)	756 (99.0)		
	Yes	26 (1.6)	8 (1.0)	0.66 (0.30- 1.47)	0.312
TCA	No	1646 (99.5)	760 (99.5)		
	Yes	8 (0.5)	4 (0.5)	1.08 (0.32- 3.61)	0.897
SSRI	No	1568 (94.8)	746 (97.5)		
	Yes	86 (5.2)	19 (2.5)	0.46 (0.28- 0.77)	0.003
PPI	No	1405 (84.7)	675 (88.5)		
	Yes	254 (15.3)	88 (11.5)	0.72 (0.56- 0.93)	0.013
Gliclazide	No	1645 (99.5)	750 (98.0)		
	Yes	9 (0.5)	15 (2.0)	3.66 (1.59- 8.39)	0.002
Insulin	No	1629 (98.4)	738 (96.5)		
	Yes	26 (1.6)	27 (3.5)	2.29 (1.33- 3.95)	0.003
Glibenclamide	No	1628 (98.4)	748 (97.8)		
	Yes	26 (1.6)	17 (2.2)	1.42 (0.77- 2.64)	0.263

Metformin	No	1541 (93.2)	687 (89.9)		
	Yes	113 (6.8)	77 (10.1)	1.53 (1.13- 2.07)	0.006
Ferrous Sulfate	No	1627 (98.2)	753 (98.6)		
	Yes	30 (1.8)	11 (1.4)	0.79 (0.39- 1.59)	0.512
Statin	No	1422 (85.9)	674 (87.3)		
	Yes	234 (14.1)	98 (12.7)	0.85 (0.66- 1.10)	0.222
ACE-I Or ARB	No	1389 (83.8)	601 (79.0)		
	Yes	269 (16.2)	160 (21.0)	1.37 (1.11- 1.71)	0.004
GnRH	No	1643 (99.3)	749 (97.9)		
	Yes	11 (0.7)	16 (2.1)	3.19 (1.47- 6.91)	0.003
Vitamin D3	No	1316 (79.4)	645 (84.8)		
	Yes	341 (20.6)	116 (15.2)	0.69 (0.55- 0.87)	0.002
Calcium	No	1361 (82.1)	654 (85.9)		
	Yes	296 (17.9)	107 (14.1)	0.72 (0.59- 0.96)	0.020

* OR is reported for inadequate groups compared with adequate groups as reference group

**Predictive model based on demographic factors**

**Figure 1:**
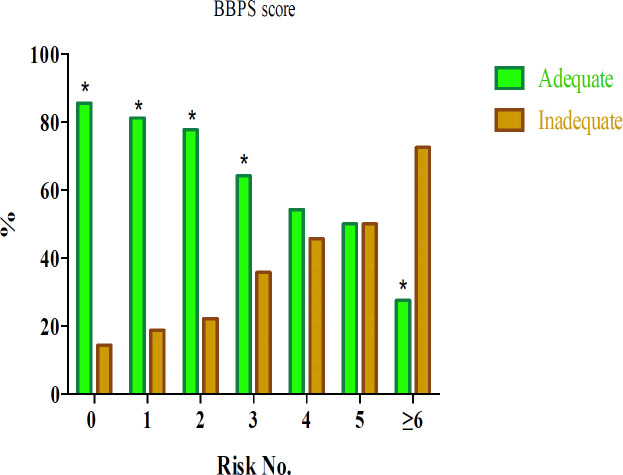
The effect of the combination of demographic factors on inadequacy of bowel preparation

**Figure 2:**
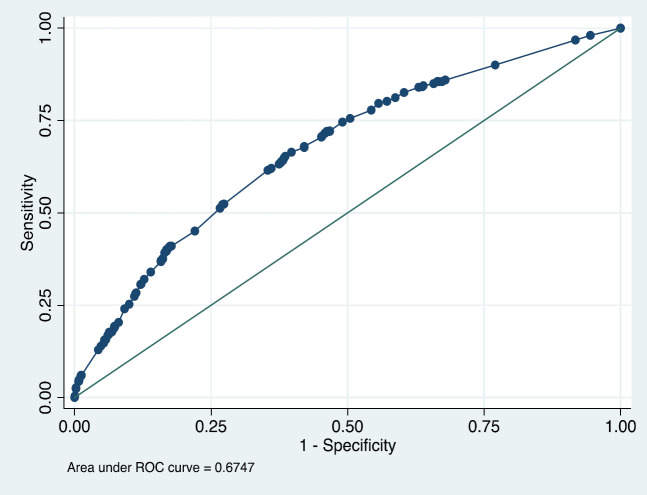
ROC curve of combined logistic model of demographic factors (including BMI, abdominal circumference, education, fruit consumption, vegetable consumption, smoking, and physical activity) (Model A)

In order to suggest a predictive model for identifying patients with a high risk of bowel preparation inadequacy, the joint assessment of the seven demographic risk factors of bowel preparation inadequacy, i.e. BMI, abdominal circumference, education, fruit consumption, vegetable consumption, smoking, and physical activity, was performed. Stepwise escalation of risk was sequentially observed for single up to over six positive subjects and yielded adjusted odds ratios ranging from 1.32 to 14.35 as compared with the sixth negative reference subjects. In the group of the patients with ≥6 risk factors, the likelihood of inadequacy was approximately 14 times greater than in patients with no risk factor, and the number of patients with inadequate bowel preparation was significantly higher than the number of patients with adequate preparation (*p*<0.001) (Figure 1). Also evaluated was the stepwise inclusion of these seven demographic risk factors in a logistic model (model A). Additionally, stepwise inclusion of each parameter to 

the model decreased the AIC and BIC and increased AUC (except for vegetable consumption) to 0.67 in the final step (Figure 2).

**Multivariate analysis of risk factors**

Demographic, anthropometric, and drug consumption data was entered into the multivariate logistic analysis and is shown in Table 4 as a crude model. The effect of age, gender, and ethnicity on colon preparation changed when these variables were simultaneously included in the model; however, the association between gender, ethnicity and other variables was found to be non-signiﬁcant (Model B, Table 4). Age (OR=1.01, 95% CI 1.01-1.02, *p*<0.0001), BMI>25 (OR=1.59, 95% CI 1.26-2.01, *p*<0.0001), abdominal circumference >95 cm (OR=1.48, 95% CI 1.17-1.87, *p*=0.001), low fruit consumption (OR=2.57, 95% CI 2.05-3.23, *p*<0.0001), and history of smoking (OR=1.34, 95% CI 1.02-1.75, *p*=0.035) were significantly correlated with bowel preparation inadequacy in the multivariate regression analysis. Furthermore, NSAIDs (OR=0.42, 95% CI 0.24-0.74, *p*=0.003) and SSRI (OR=0.40, 95% CI 0.22-0.71, *p*=0.002) were significantly correlated with bowel preparation adequacy in the multivariate regression analysis.

**Final predictive model**

The results of univariable and multivariable logistic regression analyses along with the AIC and BIC values corresponding to the inclusion/exclusion of each predictor were used to select the predictors of the full 

**Table 4:** Logistic model

Characteristic	Crude model				Model B*				Model C**			
	OR (95% CI)		P-value		OR (95% CI)		P-value		OR (95% CI)		P-value	
General characteristics												
Age (Years old)	1.01 (1.00- 1.02)		<0.0001		1.01 (1.01- 1.02)		<0.0001		1.01 (1.01- 1.02)		<0.0001***	
Gender												
Female	1				1				1			
Male	1.16 (0.98- 1.38)		0.085		1.16 (0.98- 1.38)		0.085		1.08 (0.87- 1.34)		0.465	
Ethnicity												
Fars	1				1				1			
Non-Fars	2.7 (2.1- 3.6)		<0.0001		1.08 (0.91- 1.28)		0.371		1.07 (0.88- 1.31)		0.469	
BMI												
25	1								1			
>25	2.1 (1.8- 2.5)		<0.0001						1.59 (1.26- 2.01)		<0.0001***	
Abdominal circumference												
95	1								1			
>95	2.1 (1.7- 2.5)		<0.0001						1.48 (1.17- 1.87)		0.001***	
Education												
Educated	1								1			
Illiterate	1.53 (1.19- 1.95)		0.001						1.31 (0.96- 1.78)		0.085	
Fruit consumption												
High	1								1			
Never/Low	2.33 (1.95- 2.78)		<0.0001						2.57 (2.05- 3.23)		<0.0001***	
Vegetable consumption												
High	1								1			
Never/Low	1.48 (1.24- 1.78)		<0.0001						1.11 (0.89- 1.39)		0.343	
Smoking												
Never	1								1			
Ever	1.39 (1.11- 1.74)		0.005						1.34 (1.02- 1.75)		0.035***	
Physical activity												
Never	1								1			
Ever	1.26 (1.02- 1.56)		0.030						1.14 (0.89- 1.45)		0.299	
Diseases												
History of Diabetes												
No	1								1.52 (0.92- 2.50)		0.101	
Yes	1.55 (1.12- 2.15)		0.008									
Constipation												
No	1											
Yes	1.19 (1.00- 1.43)		0.045						0.87 (0.70- 1.07)		0.188	
Drug Usage												
ASA												
No	1								1			
Yes	1.33 (1.06- 1.67)		0.014						0.96 (0.71- 1.29)		0.774	
NSAID												
No	1								1			
Yes	0.45 (0.27- 0.74)		0.002						0.42 (0.24- 0.74)		0.003***	
SSRI												
No	1								1			
Yes	0.46 (0.28- 0.77)		0.003						0.40 (0.22- 0.71)		0.002***	
PPI												
No	1								1			
Yes	0.72 (0.56- 0.93)		0.013						0.83 (0.62- 1.10)		0.199	
Gliclazide												
No	1								1			
Yes	3.65 (1.59- 8.39)		0.002						1.24 (0.38- 3.99)		0.718	
Insulin												
No	1								1			
Yes	2.29 (1.33 3.95)		0.003						1.30 (0.61- 2.75)		0.492	
Metformin													
No		1								1			
Yes		1.53 (1.13- 2.07)		0.006						1.11 (0.72- 1.70)		0.636	
ACE-I Or ARB													
No		1								1			
Yes		1.37 (1.11- 1.71)		0.004						1.19 (0.91- 1.56)		0.194	
GnRH													
No		1								1			
Yes		3.19 (1.47- 6.91)		0.003						2.07 (0.86- 5.02)		0.105	
Vitamin D3													
No		1								1			
Yes		0.69 (0.55- 0.87)		0.002						0.82 (0.61- 1.12)		0.209	
Calcium													
No		1								1			
Yes		0.72 (0.59- 0.96)		0.020						0.84 (0.61- 1.16)		0.295	

*Adjusted for Age, gender, and ethnicity; ** Adjusted for all variables within the table. ***Selected statistically significant predictors for the final model. Clinically significant predictors

**Table 5:** Full diagnostic (logistic) model for colon preparation adequacy, including the intercept (Model C)

Intercept and Predictors		Coefficient	SE	OR	95% CI	*P*-value
General characteristics						
Intercept		-2.27	0.23	-	-	
Age _(Years old)_		0.01	0.00	1.01	1.00- 1.02	< 0.0001
Gender	Female					
	Male	0.13	0.11	1.14	0.93- 1.41	0.205
Ethnicity	Fars					
	Non-Fars	0.06	0.10	1.06	0.87- 1.29	0.551
BMI	25					
	>25	0.51	0.11	1.67	1.33- 2.09	< 0.0001
Abdominal circumference	95					
	>95	0.39	0.12	1.48	1.17- 1.86	0.001
Education	Educated					
	Illiterate	0.32	0.15	1.37	1.02- 1.86	0.039
Fruit consumption	High					
	Never/Low	1.07	0.10	2.90	2.38- 3.54	< 0.0001
Smoking	Never					
	Ever	0.28	0.14	1.32	1.01- 1.72	0.041
Physical activity	Never					
	Ever	0.12	0.12	1.13	0.89- 1.43	0.315
History of Diabetes	No					
	Yes	0.50	0.19	1.65	1.14- 2.41	0.008
Constipation	No					
	Yes	-0.19	0.10	0.83	0.68- 1.01	0.060
NSAID	No					
	Yes	-0.92	0.28	0.40	0.23- 0.69	0.001
SSRI	No					
	Yes	-0.97	0.28	0.38	0.21- 0.66	0.001
LR chi2		252.19				
Hosmer-Lemshow GOF		X^2^ = 4.48, *P* value= 0.811				
ROC area (95% CI)		0.70 (0.68, 0.72)				

**Figure 3:**
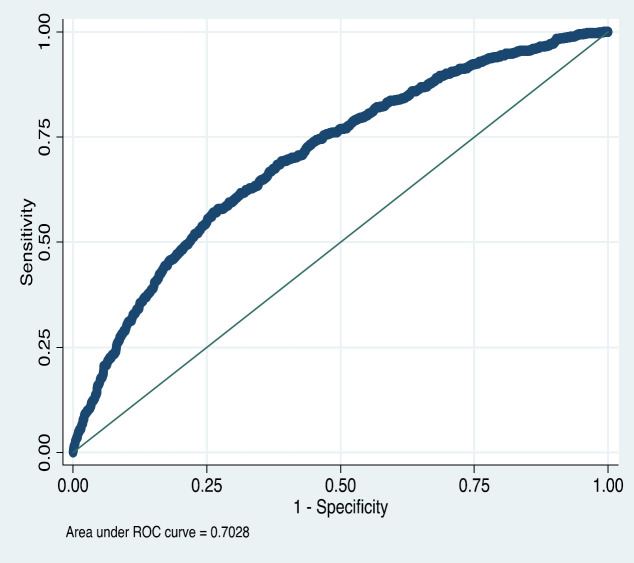
ROC curve of full diagnostic (logistic) model (Model C)

Predictor value that is one when it is present and zero when it is absent. SE: Standard error. OR: Odds Ratio. X2: Chi square statistic. GOF: Goodness of fit. ROC = Receiver-operating characteristic.

predictive (logistic) model (Model C, Table 4). The optimum model was selected by both methods corresponding with the model consisting of 13 predictors, as shown in Table 5. Additionally, the stepwise inclusion of each parameter to the model decreased AIC and BIC and increased AUC to 0.7 in the final step. Although the association of gender, ethnicity, physical activity, and education with colon preparation was not statistically significant in either univariable or multivariable analyses, they were forced into the final predictive model due to their clinical relevance. The Hosmer-Lemeshow statistic suggested that the ﬁt of the model was acceptable for the development dataset (*p* = 0.811). Figure 3 shows the ROC curves for the diagnosis of colon preparation adequacy, where the sensitivity and specificity for several risk thresholds are plotted.

## Discussion

Having appropriate bowel preparation before a colonoscopy is necessary for performing a high quality, safe, and effective procedure. In the current study, the association between bowel preparation inadequacy and demographic factors, comorbidities, and drug history was evaluated, and a predictive model is suggested for identifying those patients with a high risk of bowel preparation inadequacy before a colonoscopy to employ alternative preparation techniques for yielding optimal preparation quality.

The current results showed that 31.8% of the enrolled patients had inadequate bowel preparation before a colonoscopy, which was in concordance with previous studies reporting that bowel preparation inadequacy occurred in 17.2% to 44.2% of colonoscopies (11-16). Contrary to prior studies, however, there were no significant differences in the prevalence of preparation inadequacy between ascending, descending, and transverse colon in this study, even though several authors have described that preparation inadequacy is more frequent in ascending colon (21, 25). These controversies could be due to the difference in preparation regimens between studies, as a higher amount of PEG was used in the current study than in earlier ones.

The demographic, clinical, and drug-related factors were investigated, and it was found that age, BMI>25, and abdominal circumference >95 cm are independent risk factors for inadequate bowel preparation. In addition, patient age is considered a predictor of inadequate bowel preparation (13, 18, 19). Accordingly, impaired gastrointestinal motility, higher constipation rate (26), and a higher prevalence of comorbidities in older ages (27, 28) can be the reasons. Similar to the current findings, some previous studies confirmed that overweight and obesity are associated with bowel preparation inadequacy (13, 29). Borg et al. declared that BMI>25 is an independent risk factor of bowel preparation inadequacy, and each unit of increase in BMI could enhance the likelihood of inadequate bowel preparation by 2.1% (29). Several studies have reported that obesity (i.e. BMI>30), but not overweight (i.e. 30>BMI≥25), is associated with bowel preparation inadequacy (15, 30). This effect can be attributed to lower colonic motility and higher rates of constipation among obese patients compared to individuals with normal weight (31). The association between abdominal circumference and preparation inadequacy has not been studied previously; however, waist circumference has been reported to be positively correlated with BMI, and patients with higher adnominal circumference tend to have more BMI (32). Accordingly, this can be the reason for the association between abdominal circumference >95 cm and preparation inadequacy in the current study. Moreover, the current study found history of smoking and low fruit consumption to be independent risk factors of inadequate bowel preparation. Several studies have reported that smoking is independently associated with lower quality of bowel preparation (15, 29). Importantly, smoking can also increase colon transit time, which may explain the greater amount of residue in the colon, leading to inappropriate bowel preparation (33, 34). Although daily fruit consumption is associated with better colonic motility and lower constipation rates (35), there is no report evaluating the effect of routine dietary fibers on preparation quality. Hence, it is recommended that physicians also consider the patient’s diet. 

The impact of diabetes and constipation remains controversial in previous studies. Similar to the current study, several investigations have reported that diabetes (16, 22, 36) and constipation (13, 14, 22, 36) are not independently associated with bowel preparation inadequacy. Conversely, a considerable number of studies and two meta-analyses indicated that constipation and diabetes can significantly affect the bowel preparation quality (14, 18, 19, 21, 37, 38). The 5-HT3 receptor located in both the enteric nervous system and the central nervous system (CNS) is related to colonic peristaltic reflex, colonic motility, colonic transit, GI secretion, and sensation (39-41). Previous studies have found that serotoninergic agents can activate and increase the colonic peristaltic reflex, resulting in increased colon transit and reduced constipation (42-44). This may explain the association between these drugs and colonic preparation adequacy. No data is available on the effect of NSAIDs on colonic preparation quality; however, some authors have declared that COX-2 inhibition affects colonic smooth muscles, resulting in decreased colon transit time (45-47), which could explain the effect of NSAIDs on the enhancement of bowel preparation. Conversely, numerable studies have indicated that NSAIDs usage can cause constipation (37, 48), and therefore, decrease the bowel preparation quality. This dilemma needs further study to be accurately evaluated.

In the predictive model herein, patients with ≥6 risk factors had a chance of inadequacy approximately 14 times greater than those with no risk factor. Yaldapati et al. evaluated the effect of the number of demographic, socioeconomic, and clinical risk factors on bowel preparation inadequacy among inpatients and indicated that as the number of risk factors increased, patients were more likely to have inadequate preparation (36). In the joint assessment model for demographic risk factors in the current study, the chance of having inadequate bowel preparation was significantly higher in patients with ≥3 risk factors compared to those without any risk factors, and thus, employing alternative preparation techniques should be considered for high risk patients. The stepwise inclusion of these seven demographic risk factors in a logistic model (model A) was also evaluated, and it finally reached an AUC of 0.67 in the final step. 

To enhance the discriminatory power of this model, we suggest the final logistic predictive model (model C) comprising age, BMI>25, abdominal circumference>95 cm, low fruit consumption, and smoking by the use of NSAID and SSRI, which were independent risk factors of inadequate bowel preparation in the current study. Additionally, illiteracy (19) and diabetes (13, 18, 19, 22) have previously been described as associated risk factors with preparation inadequacy, which is in accordance with the univariate analysis performed herein. Male gender (13, 18, 20), ethnicity (non-Fars in the current study) (49), low physical activity (50-52), and constipation (18, 21, 22) were not significantly associated with preparation inadequacy in univariate and multivariate analyses; however, previous studies have indicated their relevance, and adding them to the final model increased AUC and decreased AIC and BIC. 

An AUC of 0.70 was achieved in our final model, which included thirteen factors. Gimeno-García et al. evaluated 1076 patients from a tertiary referral hospital in a development and validation cohort. The preparation regimen in their study was distinct from the current one; their regimen contained three bowel agents, sodium picosulphate plus magnesium citrate plus citric acid, PEG plus ascorbic acid, and PEG. Four independent factors that significantly affect the bowel preparation adequacy were entered into the model. The final predictive model reached an AUC of 0.70 in the validation cohort. Moreover, a scoring system was suggested to identify patients with a high risk of inadequate bowel preparation with a negative predictive value of 89.1% (21). Hassan et al. conducted a prospective multi-center study on 2811 patients and finally suggested a predictive model including eight risk factors of bowel preparation inadequacy. Their preparation method consisted of six different regimens, and the AUC of the model was 0.63 (13). Dik et al. performed a multi-center prospective study on 1996 Dutch participants who were randomly assigned into validation and development cohorts. All patients used a split-dose preparation regimen, but four different medications were used. Eventually, a logistic model comprised of eight items was proposed for predicting inadequacy in bowel preparation with an AUC of 0.77 in the validation cohort [21]. Importantly, it should be noted that preparation protocols used differed among all of these previous studies and the current one. In the present study, the patients who underwent a morning colonoscopy using a single-dose preparation regimen, and those undergoing an evening colonoscopies used a split-dose preparation regimen. A higher volume of PEG was used compared to the mentioned studies (6 liters of PEG+3 tablets of bisacodyl). 

The proposed model has a fundamental difference from those in previous studies; in addition to independent risk factors of bowel preparation inadequacy, risk factors that were significantly associated with bowel preparation inadequacy in previous studies or were associated only with preparation inadequacy in univariate analysis were entered into the model. However, increased AUC in the stepwise inclusion enhanced the discriminative ability of the final model. The AUC of the proposed model was similar to that of previous studies [9, 20, 21], which showed fair discriminative ability.

The strengths of the current study are: (1) large population from a gastroenterology university center; (2) complete evaluation of drug associations with bowel preparation inadequacy (which has not been completely studied); and (3) entering the factors that were significantly associated with bowel preparation inadequacy in previous studies in addition to independent predictors of preparation inadequacy in the current study to improve the generalizability and discriminative abilities of the predictive model. 

However, some limitations of this study need to be addressed. First, this study suggests a predictive model for bowel preparation inadequacy, but no validation study was done for the predictive rule. Second, the discriminative ability of the proposed model is moderate. Finally, no validated score was suggested to predict the preparation inadequacy in the current study.

The current study identified some independent risk factors that are associated with bowel preparation inadequacy, and accordingly, a predictive model for identifying patients with a high risk of inadequate bowel preparation before colonoscopy is suggested. Identifying and eliminating reversible causes of inadequate preparation, alternative cleansing techniques, and intensified preparation protocols are necessary for these patients to reach acceptable preparation adequacy before colonoscopy. Further studies are needed to validate the model, or to suggest a model with higher discriminative ability, and to invent a predictive score for identifying patients with a higher risk of bowel preparation inadequacy.

## Conflict of interests

The authors declare that they have no conflict of interest.
